# Validation of an Estonian version of the Parkinson's Disease Questionnaire (PDQ-39)

**DOI:** 10.1186/1477-7525-6-23

**Published:** 2008-03-25

**Authors:** Ülle Krikmann, Pille Taba, Taavi Lai, Toomas Asser

**Affiliations:** 1 Department of Neurology and Neurosurgery, University of Tartu, Tartu 51014, Estonia; 2 Department of Public Health, University of Tartu, Estonia

## Abstract

**Introduction:**

Diagnosis and management of Parkinson's disease (PD) rely heavily on evaluation of clinical symptoms and patients' subjective perception of their condition. The purpose of this study was to evaluate the validity, acceptability, and reliability of the Estonian version of the 39-question Parkinson 's disease Questionnaire (PDQ-39).

**Methods:**

Study subjects were approached during their regular clinic follow-up visits. 104 patients consented to the study and 81 completed questionnaires were used for subsequent testing of psychometric characteristics, validity and reliability.

**Results:**

The content validity was assessed through qualitative content analysis during the pilot study. The patients indicated that the questions were relevant to measure the quality of life of people with PD.

The analysis of means showed that the ceiling and floor effects of domain results were within the limits of 15% of Summary Index and of all domains except Stigma, Social Support and Communication where the ceiling effect was 16% to 24% of the responses. Convergent validity was interpreted through correlation between disease severity and PDQ-39 domains. There was a statistically significant difference between the domain scores in patients with mild versus moderate PD in domains of Mobility, ADL, and Communication but not for Stigma, Social Support and Cognition. The reliability was good, Cronbach alpha for all domains and summary index was over 0.8 and item-test correlations between domains and summary index ranged from 0.56 to 0.83.

**Conclusion:**

The psychometric characteristics of an Estonian version of the PDQ-39 were satisfactory. The results of this study were comparable to the results of previous validation studies in other cultural settings in UK, USA, Canada, Spain and Italy.

The Estonian version of the PDQ-39 is an acceptable, valid and reliable instrument for quality of life measurement in PD patients.

## Introduction

Parkinson's disease (PD) is a progressive neurodegenerative disorder, characterised by bradykinesia, tremor, disturbances of postural reflexes and of the autonomic nervous system, and which is most prevalent in old age [1-3].

Diagnosis and management of PD is based on clinical assessment of symptoms and signs using disease specific rating scales [4,5]. Evaluation of disease severity, effect of treatment procedures and interventions are all influenced by the patient's perception of their disease and assessment instruments have to take this aspect into account [4-6]. Such instruments measuring different factors affecting patient's perception of well-being in relation to disease and health are referred to as health related quality of life (HRQOL) questionnaires. Measures of general HRQOL, like the SF-36 [7,8] are used widely but are less suited to assessment and management of a specific health condition compared to their disease specific counterparts, like the 39-question Parkinson's Disease Questionnaire (PDQ-39) [9].

The PDQ-39 has been translated into many languages all over the world and extensively used for PD research by many authors [10-13]. Translation and validation of an instrument is a prerequisite for its use in a new cultural context and only after validation is it possible to compare the research data in a cross-cultural context [14,15].

The authors hypothesized the Estonian version PDQ-39 to be an acceptable, valid and reliable measure for use in PD patients in Estonia and the aim of current study was to test these hypotheses.

## Methods

### Questionnaire translation

The PDQ-39 consists of 39 questions, distributed between eight multi-item domains: Mobility – ten questions; Activities of Daily Living – six questions; Emotional Well-being – six questions; Stigma – four questions; Social Support – three questions; Cognition – four questions; Communication – three questions; Bodily Discomfort – three questions. Responses to all questions are coded and mapped to a percentage scale where 0 denotes "no problem" and 100 "maximum level of problem". Finally domain scores are generated by calculating a simple average over all domain item scores [6,9].

The translation procedure in our study took into account different guidelines, recommendations and examples previously published on translation and cultural adaptation of quality of life measures [11-16]. The procedure comprised forward translation, assessment of item comprehension, back translation to English and development of a consensual version based on the results of previous translations and comprehension assessments. First, forward translation of the original PDQ-39 was independently carried out by two native Estonian speakers with excellent knowledge of English. Reconciliation of these two translations was then performed, followed by back translation into English by a third translator without access to the original version of PDQ-39. Comparison of the back-translation and original versions of the questionnaire was performed and the results were used for the development of the final consensual translation of the Estonian language PDQ-39. Distance conversion into the metric system was performed as a part of the translation process when questionnaire items involved such measurements.

### Patients

The study included pilot-testing of the final Estonian version of the PDQ-39 using a group of 15 PD patients from the Tartu Parkinson's Disease Society [17]. All the patients had first-hand knowledge of PD and agreed to comment on the understandability and relevance of the questionnaire items during a group interview based on focus group methodology. Descriptive figures on the group composition are given in the Table 1.

**Table 1:** Comparison of the characteristics of responders and non-responders

	Pilot study for qualitative assessment	Main study for psychometric statistical analysis	
	n = 15	Responders n = 81	Non-responders n = 23

Female/male (N)	9/6	55/26	10/11
Mean age, years (SD)	71.4 (8.9)	66.9 (8.2)	72.3 (7.4)
Age range	64–86	48–85	52–86
Duration of disease, years (SD)	7.8 (10.3)	8.9 (6.3)	7.5 (9.3)
Range	1–34	1–35	1–36
Treatment with levodopa (N of patients)	11	74	18
Combination antiparkinsonian medications (N of patients)	12	48	4
Adverse effects (dyskinesias, fluctuations etc) (N of patients)	12	44	4
Severity of disease HY I–II	5	48	15
HY III	7	29	7
HY IV–V	3	4	1

The main study involved 108 patients with a previous diagnosis of idiopathic PD who were approached during their regular follow-up visits to the neurological out-patient clinic of the Department of Neurology and Neurosurgery of the University of Tartu which is one of the two tertiary healthcare providers in Estonia. Patient recruitment was also carried out in 5 regional secondary level out-patient centres (out of 8) during 1999–2001 for a random mix of complex and regular cases. All patients consented to initial medical examination by the first author. Patients satisfying the criteria of the UK PD Society Brain Bank [18] and scoring over 25 points (out of 30) on cognitive function testing using the Mini Mental State Examination (MMSE) [19] were proposed to take part in the study. One hundred and four patients satisfied the inclusion criteria, completed informed consent forms and were enrolled into the study.

Disease severity assessment was performed during the recruitment visit, using the Unified Parkinson's Disease Rating Scale (UPDRS version 3.1) and Hoehn and Yahr (HY) staging system [4,5]. All assessments of cognitive ability and disease severity were conducted when the patient's health status was stable and PD was in the 'on' period. Patients were grouped by severity of disease as follows: mild (HY stages I–II), moderate (HY III) and severe disease (HY IV–V). Disease grouping was used in order to estimate the correlation between clinical stages and PDQ-39 domains for assessment of convergent validity.

Co-morbidities were ascertained using medical records (e.g. depression was recorded as a co-morbid condition in case medical records included indications of depression, sleep disorder or antidepressant prescription). In addition, current medications for both PD and other diseases along with treatment side effects were recorded during the first examination.

PDQ-39 questionnaires were handed to study subjects at the end of their recruitment visit for self-reporting in their usual environment. The collection period for returned questionnaires was 4 weeks. Second copies of PDQ-39 (responses of which were not used for other psychometric tests) were mailed to all patients two weeks after the reception of the first questionnaire. The PDQ-39 was accompanied by a checklist on health status change during these two weeks. This test-retest was carried out to assess the reproducibility of the PDQ-39 results.

### Acceptability, validity and reliability

The psychometric analysis of the Estonian version of the PDQ-39 was performed by evaluation of acceptability, validity, and reliability of the questionnaire using qualitative content analysis and a variety of statistical procedures.

All PDQ-39 domains and general health status data were checked for floor and ceiling effects and less than 15% of minimum or maximum values per domain were considered acceptable [20]. Skewness and kurtosis were calculated to ascertain normal distribution of the data. Background data were analysed using univariate methods and ANOVA. The Kruskal-Wallis test was applied to compare the distribution of mean scores of the domains of PDQ-39.

Content validity of the questionnaire was tested during the pilot-testing of the PDQ-39 translation. Group discussion and interviews followed completion of the questionnaire regarding how easy it was to understand, whether it measured quality of life and how relevant it was.

Construct validity was assessed by comparing PDQ-39 domain scores by the HY stages using ANOVA statistics.

Cronbach alpha greater than 0.7 was considered a strong indication of questionnaire reliability. Item-total correlations over 0,4 were considered to show acceptable correlation between the questionnaire items. Spearman rank correlation was used to show item-item correlations.

Reproducibility was tested using test-retest analysis and a correlation of 0.7 or more determined an adequate result. All test results with an error level of 5% or less (*p *≤ *0.05*) were considered statistically significant.

## Results

Psychometric analysis was conducted in two groups of patients: 15 patients in the pilot study for processing the qualitative assessment of the questionnaire and 104 patients in the main study group, for which 81 self-reported questionnaires were completed and used in the statistical analysis, a response rate of 79%. The PD patients who entered the final analysis group were categorised by severity of disease as follows: 48 patients (59%) in mild stage (HY stage I–II), 29 patients (36%) in moderate stage (HY III) and 4 patients (5%) in severe stage (HY IV–V). Non-responders belonged to mild (16 patients) and moderate (5 patients) disease groups while two disease severity assessment questionnaires were unusable. Descriptive characteristics of the responders and non-responders are shown in Table 1.

The most frequently reported concomitant diseases were high blood pressure (24%), coronary heart disease (24%), and joint disease (14%). There were two cases of stroke and one of malignancy among the patients in the severe disease group. The majority (60%) of patients lived in an urban area. The most common medication was levodopa and all patients with severe PD suffered from adverse effects of their medications.

**Figure 1:**
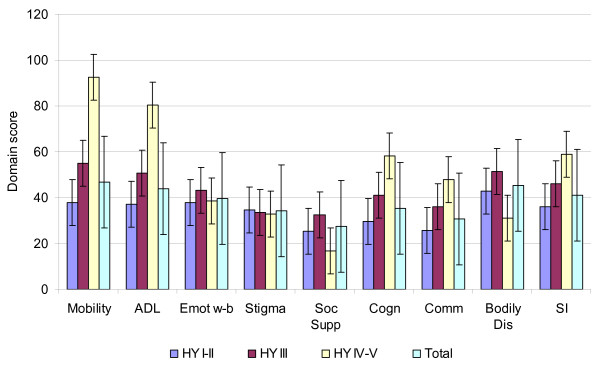
**Mean scores and standard errors of PDQ-39 domains by Hoehn and Yahr stages of illness**. ADL-Activities of Daily Living; Emot w-b - Emotional Well-being; Soc Sup - Social Support; Cogn - Cognition; Comm - Communication; Bodily Dis -  Bodily Discomfort; SI – Summary Index.

Validity interpretation was carried out using the ability of the PDQ-39 to distinguish high and low symptom burden and correlation of questionnaire responses to clinically assessed severity of PD. The patients in mild stages of PD had an average summary index score 35.8, for patients in a moderate stage of disease it was 46.1 and for patients in an advanced stage of the disease it was 59.1 (Figure 1). There was a statistically significant difference between the domain scores in patients with mild versus moderate PD for the domains of Mobility, ADL, and Communication. The difference in scores for Stigma, Social support and Cognition was not significant.

Content validity was assessed through the qualitative content analysis during the pilot study. All the questions and domains were essential to PD patients for measurement of quality of life.

Internal consistency for all domains was good as the Cronbach alpha measure was above 0.7 in all cases (ranging from 0.81 to 0.86) indicating good reliability of the questionnaire (see details in Table 2). Item-test correlations for all questions and domains ranged from 0.56 to 0.83 again confirming the consistency of the questionnaire (only domain figures are given in Table 2 for the sake of brevity). The item-item correlations of mean domain scores were moderate ranging from 0.67 between Mobility and Activities of Daily Living to 0.2 between Communication and Bodily Discomfort (Table 3) as expected.

**Table 2:** Internal consistency (Cronbach alpha) and item-test correlations between mean domain scores and the summary index score of the PDQ-39 (n = 81).

Domain	Cronbach alpha	Item-test correlation
Mobility	0.85	0.69
Activities of daily living	0.85	0.72
Emotional well-being	0.85	0.83
Stigma	0.86	0.72
Social support	0.84	0.68
Cognition	0.82	0.67
Communication	0.83	0.56
Bodily discomfort	0.81	0.69

All the coefficients were significant (*p *< 0.05).

**Table 3:** Spearman rank correlation between domains and summary index (SI) of the Estonian version PDQ-39.

	Mobility	ADL	Emot w-b	Stigma	Soc supp	Cogn	Comm	Bodily dis
Mobility								
Activities of daily living (ADL)	0.67							
Emotional well-being (Emot w-b)	0.51	0.45						
Stigma	0.30	0.37	0.57					
Social support (Soc supp)	0.32	0.37	0.66	0.53				
Cognition (Cogn)	0.43	0.49	0.49	0.29	0.35			
Communication (Comm)	0.32	0.32	0.25	0.28	0.26	0.43		
Bodily discomfort (Bodily dis)	0.34	0.34	0.61	0.43	0.52	0.42	0.20	
Summary index (SI)	0.81	0.79	0.8	0.62	0.66	0.64	0.64	0.46

Reproducibility of the results and test-retest reliability of the questionnaire were assessed by correlation between results of test and retest questionnaires from a selection of patients. These results ranged from 0.72 to 0.92 indicating good reproducibility. The retest number of responders was 78.

The whole range of possible scores (0–100) of the PDQ-39 domains were covered. The mean and median were very similar in the domains of Mobility, Activity of Daily Living (ADL), Emotional Well-Being and Stigma, with a difference of less than 10%. For Cognition, the difference was 16%. The difference between the mean and median for the summary index was approximately 1% (see Table 4 for details).

**Table 4:** Mean, standard deviation (SD) median scores, 95% confidence interval, skewness and kurtosis of domains of the PDQ-39 Estonian version (n = 81).

Domain	Mean	SD	Median	95% Confidence interval	Skewness	Kurtosis
Mobility	46.7	26.1	47.5	40.9 – 52.5	0.1	2.3
Activities of daily living	44.1	25.1	41.7	38.5 – 49.6	0.3	2.1
Emotional well-being	39.8	25.0	37.5	34.3 – 45.3	0.3	2.1
Stigma	34.2	28.3	31.3	27.9 – 40.3	0.5	2.2
Social support	27.6	24.5	25.0	22.1 – 32.9	0.8	3.3
Cognition	35.2	21.8	41.7	37.4 – 46.4	0.1	2.2
Communication	30.6	20.8	33.3	25.9 – 35.2	0.3	3.1
Bodily discomfort	45.3	22.5	50.0	40.3 – 50.3	0.1	2.0
Summary index	39.0	17.7	38.6	36.7 – 44.6	0.1	2.4

The floor effect ranged from 1.2% to 2.5% in the domains of Mobility, ADL, Emotional Well-being, Cognition and Bodily Discomfort, while for the domains of Stigma, Social Support and Communication it ranged from 16% to 24%, which was more than hypothesized. It is also noteworthy that among patients with moderate or severe PD patients the response distribution was more central and the only floor effect was recorded for the Social Support domain (21% compared to the 25% in the mild PD group). The lowest ceiling effects (1.2%) were observed in Mobility, ADL, Emotional Well-Being, Social Support and Communication domains. In other domains the ceiling effect was between 2.4% to 3.7%. The criterion of ceiling effects below 15% was achieved in all domains. Both ceiling and floor effects of the Summary index (SI) were at the 1.2% level. The analyses showed that the distribution of the results in the domains and summary index was within the limits of the preset criteria in most cases.

## Discussion

This paper presented the evidence on acceptability, validity and reliability of the Estonian version of the PDQ-39 which were good as originally hypothesised. The descriptive statistics for the acceptability assessment of the questionnaire showed a good distribution of the response choices on the scale level, ceiling and floor effects were low and within hypothesized limits with only few exceptions. Content and face validity tests unambiguously showed that the covered topics and items in the translated PDQ-39 questionnaire are relevant to the Estonian PD patients. Validity assessment of the Estonian version of the PDQ-39 was guided by experience from previous similar studies [6,21-25] and as expected the localised version of PDQ-39 was able to differentiate between disease groups with the lowest and highest PD burden. Most of the previous validation studies in other cultural settings [9-16] have used a variety of rating scales and classifications like Hoehn and Yahr stages, UPDRS scores and main symptoms for assessment of construct validity. All these studies found statistically significant differences between disease severity and PDQ-39 domains as an example for the domains of Mobility and Activities of Daily Living (mean scores ranging from 22.34 (HY I) to 75.25 (HY IV–V) [6,22,24,25] which ranged from 36.5 (HY I–II) to 90.5 (HY IV–V) in our study. The differences of the domains of Stigma, Social support, Emotional Well-Being and Bodily discomfort were not so clearly related to the severity of PD stages. The summary index scores were the lowest in the patient group with mild PD and highest in the severe disease group as expected to once more indicating close psychometric values to other language versions of PDQ-39 [11,12,22]. The somewhat worse domain scores especially in the case of Mobility and Activities of Daily living could be related to the still developing social care system in Estonia but verification of this assumption would recuire a direct comparison of country situations and respective social care systems.

Regarding the reliability and other internal consistency measures the strongest correlations between domain and total questionnaire score have been found for the domains of mobility and Activities of Daily Living [9-16]. These two domains showed strong item-test correlations in our study as well. The reliability figures of Estonian version of PDQ-39 surpassed the results from cross-cultural studies from USA, Canada, Spain, Italy and Japan [10] as witnessed by Cronbach alpha above 0.80 [26] for all domains compared to a wider range (from 0.13 to 0.96) in those studies. The lowest Cronbach alpha coefficients in those studies were found in Japan and seemed to be connected to that particular cultural background [10]. Test-retest analysis showed no differences of score distributions between the two assessments as no changes were recorded in previous reports of the original version of PDQ-39 [15,22,24]. The localisation of PDQ-39 to Estonian context was successful since all preset criteria were met and the results of this study were comparable to the results of previous validation studies in other cultural settings in UK, USA, Canada, Spain and Italy.

One of the possible drawbacks of the current study is associated with the relatively small sample size. However, during the study planning a relative size of the study group was set at 5% of the total PD population in Estonia and that target was reached with the current sample. Also, the mean age of the patients in the sample corresponded well to the mean age of the overall PD population in Estonia [27]. Especially good representativeness of the Estonian PD population was achieved for the patient groups with mild and moderate disease. However, the patient group with severe PD had better response rate than the group with mild PD as 4 out of 5 patients did return a filled questionnaire. The PDQ-39 is a self-administered written questionnaire and the study inclusion criteria therefore were set to include only patients having the ability to perform that particular task. The small number of patients with severe PD available for validation of written PDQ-39 highlighted the need for a HRQL measure in Estonia that could be administered in an interview form to the severe patients of PD.

The analysis of sample variation and statistical significance testing showed that albeit the possible drawbacks related to the sample size and composition the Estonian language version of PDQ-39 performed well in the psychometric testing. Therefore, the use of PDQ-39 is highly warranted for disease assessment and treatment management among the PD patients able to administer the questionnaire as the localised version of the PDQ-39 is a valid and reliable tool in this setting.

## Conclusion

The Estonian version of the PDQ-39 demonstrated similar validity and reliability to the original English language version.

It is an acceptable, valid and reliable instrument to measure quality of life of PD patients in future studies in Estonia.

## Abbreviations

PD – Parkinson's disease; PDQ-39 – Parkinson 's disease Questionnaire; HRQOL – health related quality of life; ADL – Activities of Daily Living; MMSE – Mini Mental State Examination; HY – Hoehn and Yahr staging system; UPDRS – Unified Parkinson's Disease Rating Scale version 3.1.

## Competing interests

The author(s) declare that they have no competing interests.

## Authors' contributions

ÜK collected data and drafted the whole manuscript. TA was involved in conception and design the study. PT contributed in interpretation of data and in selection of patients, TL performed statistical analysis and contributed in interpretation of data. All authors read and approved the final manuscript.
